# Functional characterisation of human pulmonary monocyte-like cells in lipopolysaccharide-mediated acute lung inflammation

**DOI:** 10.1186/1476-9255-11-9

**Published:** 2014-03-31

**Authors:** Mairi Brittan, Laura C Barr, Niall Anderson, Andrew Conway Morris, Rodger Duffin, John A Marwick, Fiona Rossi, Shonna Johnson, Kev Dhaliwal, Nikhil Hirani, Adriano G Rossi, A John Simpson

**Affiliations:** 1 BHF/University Centre for Cardiovascular Science, University of Edinburgh, Edinburgh, UK; 2 Medical Research Council Centre for Inflammation Research, University of Edinburgh, Edinburgh, UK; 3 Centre for Population Health Sciences, The University of Edinburgh Medical School, Edinburgh, UK; 4 Institute of Cellular Medicine, Medical School, Newcastle University, Newcastle upon Tyne, UK; 5 BHF Centre for Cardiovascular Science, Scottish Centre for Regenerative Medicine, The University of Edinburgh, 5 Little France Drive, Edinburgh EH16 4UU, UK

**Keywords:** Monocytes, Macrophages, Acute lung inflammation, Lipopolysaccharide, Multiparameter flow cytometry, Corticosteroid

## Abstract

**Background:**

We have previously reported the presence of novel subpopulations of pulmonary monocyte-like cells (PMLC) in the human lung; resident PMLC (rPMLC, HLA-DR^+^CD14^++^CD16^+^cells) and inducible PMLC (iPMLC, HLA-DR^+^CD14^++^CD16^-^ cells). iPMLC are significantly increased in bronchoalveolar lavage (BAL) fluid following inhalation of lipopolysaccharide (LPS). We have carried out the first functional evaluation of PMLC subpopulations in the inflamed lung, following the isolation of these cells, and other lineages, from BAL fluid using novel and complex protocols.

**Methods:**

iPMLC, rPMLC, alveolar macrophages (AM), neutrophils, and regulatory T cells were quantified in BAL fluid of healthy subjects at 9 hours post-LPS inhalation (n = 15). Cell surface antigen expression by iPMLC, rPMLC and AM and the ability of each lineage to proliferate and to undergo phagocytosis were investigated using flow cytometry. Basal cytokine production by iPMLC compared to AM following their isolation from BAL fluid and the responsiveness of both cell types following *in vitro* treatment with the synthetic corticosteroid dexamethasone were assessed.

**Results:**

rPMLC have a significantly increased expression of mature macrophage markers and of the proliferation antigen Ki67, compared to iPMLC. Our cytokine data revealed a pro-inflammatory, corticosteroid-resistant phenotype of iPMLC in this model.

**Conclusions:**

These data emphasise the presence of functionally distinct subpopulations of the monocyte/macrophage lineage in the human lung in experimental acute lung inflammation.

## Background

We have recently published data resulting from a randomised controlled trial (RCT) investigating the effects of mononuclear cell depletion by leukapheresis in healthy volunteers following lipopolysaccharide (LPS)-mediated acute lung inflammation [1]. This RCT permitted further investigation into the properties of novel subpopulations of pulmonary monocyte-like cells (PMLC) present in the human lung, as recently reported by our laboratory [2]. PMLC are subcategorised into resident (rPMLC; HLA-DR^+^CD14^++^CD16^+^) and inducible (iPMLC; HLA-DR^+^CD14^++^CD16^-^) subpopulations, the latter being significantly increased in bronchoalveolar lavage (BAL) fluid following inhalation of LPS, in parallel with a significant increase in neutrophils, and a significant decrease in regulatory T cells [2]. Monocyte-like cells have been described in the human lung, with an expansion of this population in inflammatory lung diseases [3-5]. We herein report the first functional evaluation of subpopulations of monocyte-like cells present in the inflamed lung, following the isolation of these cells, and other lineages, from BAL fluid using novel and complex protocols. A clearer understanding of PMLC is important given their possible regulatory role in acute lung inflammation and their potential as a cellular target for future therapies. Our original expectation was that iPMLCs may play a role in coordinating inflammatory cell recruitment to the lung during acute inflammation, and in orchestrating the subsequent course of the inflammatory response. We speculated that, at least at the time point studied here, iPMLCs may exhibit a relatively pro-inflammatory functional response.

We therefore quantified iPMLC, rPMLC, alveolar macrophages (AM), neutrophils, and regulatory T cells present in BAL fluid at 9 hours post-LPS inhalation in control subjects who participated in our RCT (i.e. those subjects who inhaled LPS and were then randomised to receive “sham” leukapheresis, n = 15). Characterisation of cell surface antigen expression by iPMLC, rPMLC and AM using polychromatic flow cytometry, and the ability of each lineage to proliferate and to undergo phagocytosis were investigated. We also assessed basal cytokine production by iPMLC compared to AM following their isolation from BAL fluid using fluorescence-activated cell sorting (FACS), and the responsiveness of both cell types following *in vitro* treatment with the synthetic corticosteroid dexamethasone.

## Methods

### Subjects and study protocol

Details of the RCT including subject demographics are described elsewhere [1]. For this component of the study, healthy male non-smokers aged 18-40 (n = 15) inhaled 60 μg LPS (from *Escherichia coli* 026:B6, L2654; Sigma, Poole, UK) using an inhalation-synchronised dosimeter nebuliser (Spira Elektro 2, Hameenlinna, Finland) as described [6], and were then randomised to receive sham leukapheresis of 4 total blood volumes 2 hours later using the COBE SPECTRA Apheresis System MNC Program (version 4.7) (CaridianBCT, Lakewood, CO, USA). Bronchoscopy and BAL were performed at 9 hours [2,6].

This research was carried out in compliance with the Helsinki Declaration and was approved by the Lothian research ethics committee and the NHS Lothian/University of Edinburgh Research and Development Office, Edinburgh, UK.

### Flow cytometry

BAL fluid cells were washed in phosphate-buffered saline (PBS) and resuspended at 1 × 10^6^ cells/ml in 0.1% bovine serum albumin (BSA) in PBS with Ca^2+^/Mg^2+^. Cells (1 × 10^5^ per sample) were incubated for 40 minutes at 4°C with antibodies against specific cell surface antigens (Table 1). Unstained cells, single antibody stains and fluorescence minus-one (FMO) controls were used. Erythrocytes were lysed using FACS Lysing Solution (BD Biosciences, San Jose, CA, USA) (20 minutes, 20°C). Samples were washed in PBS, centrifuged and resuspended for analysis using a BD SORP LSRFortessaII cell analyser with FACSDiva software (BD Biosciences). Anti-mouse Ig BD CompBeads™ were used to calculate automatic compensations; these were also verified manually for each antibody. FlowJo version 9.2 (TreeStar, OR, USA) was used for data analysis.

**Table 1:** Primary antibodies used for flow cytometry and FACS

**Fluorochrome-conjugated antibody**	**Clone**	**Manufacturer, catalogue number**	**Laser filter, bandpass**
**PMLC and Macrophages**			
Anti-human (h)CD14 PerCP/Cy5.5	HCD14	BioLegend, 325622	Blue, 695/40
Anti-hCD16 PE/Texas Red	3G8	Invitrogen, MHCD1617	Yellow/green (Y/G), 610/20
Anti-hHLA-DR V450	G46-6	BD Horizon, 561359	Violet, 450/50
Anti-hCD206 PE	15-2	BioLegend, 321105	Y/G, 582/15
Anti-hCD163 APC	GHI/61	BioLegend, 333609	Red, 670/30
Anti-hKi-67 PE	Ki-67	BioLegend, 350504	Y/G, 582/15
Anti-hCD71 FITC	CY1G4	BioLegend, 334104	Blue, 530/30
Anti-human mature macrophage marker (25 F9) APC	eBio25 F9	eBioscience, 14-0115-82	Red, 670/30
**Regulatory T cells**			
Anti-hCD3 FITC	UCHT1	BD Pharmingen, 555332	Blue, 530/30
Anti-hCD4 APC/Cy7	RPA-T4	BioLegend, 300518	Red, 780/60
Anti-hCD25 PE	BC96	BioLegend, 302606	Y/G, 582/15
Anti-hCD127 PE/Cy5	eBioRDR5	eBioscience, 15-1278-42	Y/G, 670/30

Staining panels were prepared using specific combinations of antibodies in order to analyse multiple cell subpopulations within a single sample. *FITC = fluorescein isothiocyanate; PE = phycoerythrin; APC = allophycocyanin.*

### Fluorescence activated cell sorting (FACS) and dexamethasone treatment of AM and iPMLC ***in vitro***

In order to isolate iPMLC and AM from BAL fluid using FACS, the remaining BAL cells were incubated with the relevant antibodies (Table 1) for 30 minutes at 4°C, washed in PBS and resuspended in 1% autologous serum in PBS. A BD FACSAriaII cell sorter was used with FACSDiva software (BD Biosciences). Sorted cells were collected into 10% autologous serum in PBS and a small proportion was analysed to assess the purity of the isolated population. Samples of less than 95% purity were discarded. Cells were incubated under standard cell culture conditions with and without 100 nM dexamethasone for 24 hours. Cell supernatants were extracted and a BD Cytometric Bead Array (BD Biosciences, San Diego, CA, USA) was used to measure the concentration of a number of cytokines.

### Phagocytosis

pHrodo™ Red *E. coli* BioParticles® (Life Technologies, UK) were incubated with *E. coli* BioParticles® opsonising reagent (Life Technologies, UK) for 1 hour (37°C) and then washed twice in PBS using centrifugation for 5 minutes at 300 g (4°C). BAL cells were placed on ice for 10 minutes and were then incubated for 15 minutes at 4°C or 37°C with either the opsonised *E. coli* BioParticles® or PBS control, as *per* the manufacturer’s instructions. Following the recommended lysis and wash steps, the samples were re-suspended in FACS Lysing Solution and analysed using a BD SORP LSRFortessaII cell analyser.

### Statistical analyses

GraphPad Prism (version 6, GraphPad Software Inc., CA, USA) was used for data analysis. Normally distributed data were analysed by unpaired *t*-test with Welch’s correction and expressed as mean (SD). Data that were not normally distributed were expressed as median [interquartile range] and were analysed by Mann Whitney *U* test. A *P* value of <0.05 was considered significant.

## Results

### Cellular composition of BAL fluid

**Figure 1:**
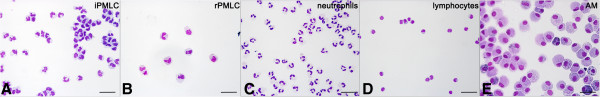
**Cytocentrifuge preparations of cells in BAL fluid following LPS inhalation.** iPMLC **(A)**, rPMLC **(B)**, neutrophils **(C)**, lymphocytes **(D)** and alveolar macrophages (AM) **(E)** were isolated from BAL fluid with high purity using FACS. PMLC are similar in morphology to blood monocytes and are a similar size to neutrophils, larger than lymphocytes and smaller than AM (Scale bar = 20 μm). Typical morphological features of each lineage were observed i.e. multi-lobular neutrophils, lymphocytes with high nucleus to cytoplasm ratio, and “fried egg”-shaped AM.

**Figure 2:**
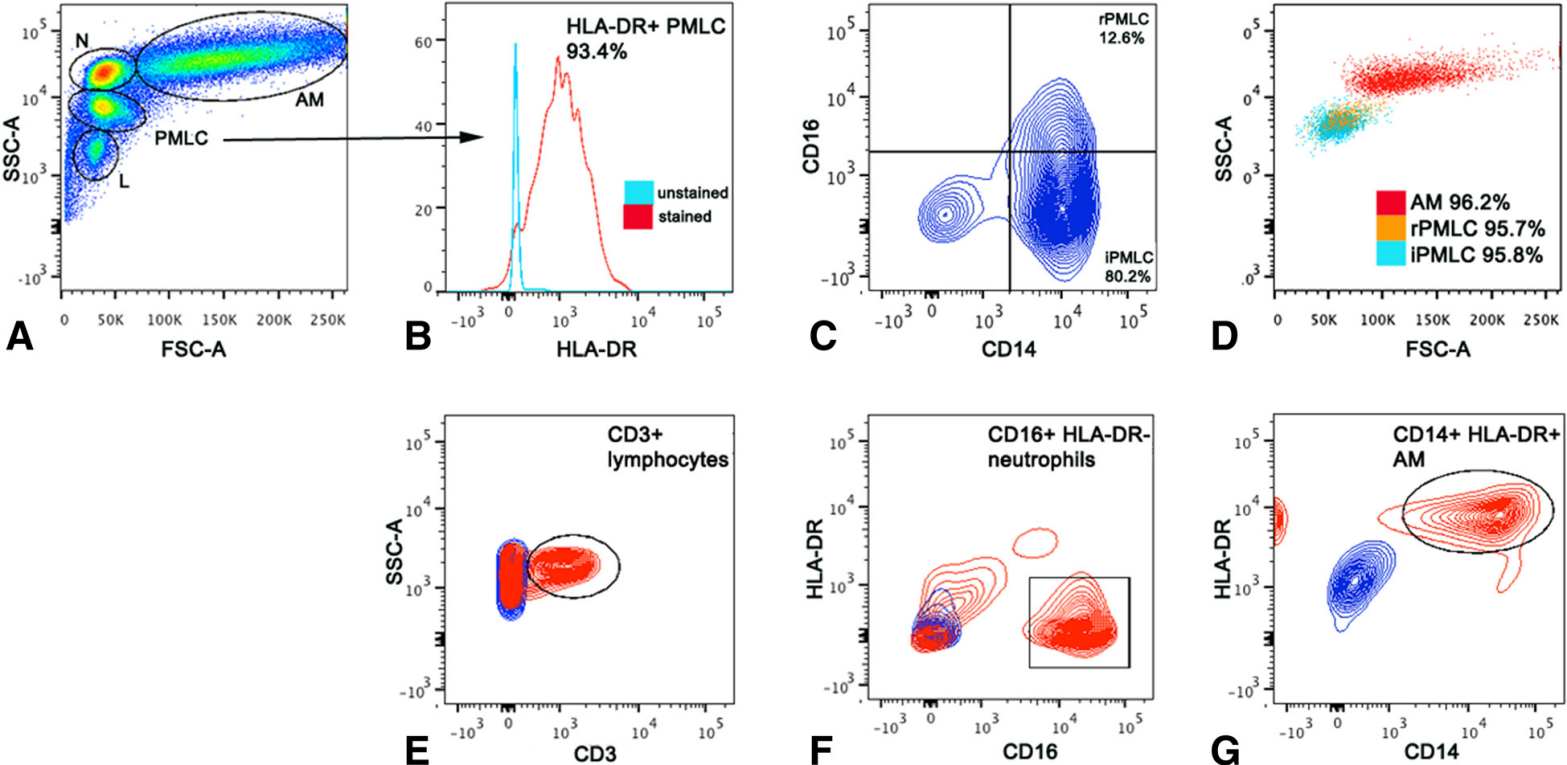
**Fluorescence activated cell sorting of cells in BAL fluid following LPS inhalation.** Cells were identified in BAL fluid based upon their position on flow cytometry dot plots of size i.e. forward scatter (FSC-A) versus granularity i.e. side scatter (SSC-A) **(A)**. PMLC were selected as HLA-DR + **(B)**, and were subdivided into iPMLC and rPMLC subpopulations based upon their CD14 and CD16 expression (CD14++CD16- and CD14++CD16+, respectively; **C**). Representative samples of AM, iPMLC and rPMLC isolated from BAL fluid using FACS with >95% purity, and overlapped on SSC-A versus FSC-A dot plots are seen in **(D)**. Like PMLC, it was possible to identify distinct populations of lymphocytes, neutrophils and alveolar macrophages in BAL fluid following LPS inhalation based upon their size and granularity **(A)**. Lymphocytes were further selected for CD3 expression **(E)**, neutrophils were classed as CD16+ HLA-DR- **(F)** and AM were selected as large CD16+ HLA- DR + cells **(G)**.

One subject did not undergo bronchoscopy and BAL as his forced expiratory volume in 1 second (FEV_1_) had fallen by >10% from baseline (a pre-defined safety criterion). Figure 1 shows cytocentrifuge preparations of iPMLC, rPMLC, neutrophils, lymphocytes, and AM isolated from BAL fluid with greater than 95% purity by FACS, in order to investigate the morphology and size of each cell population. The gating strategies used to identify cell subpopulations within BAL fluid using flow cytometry and for FACS isolation are outlined in Figure 2. The proportion of iPMLC (HLA-DR^+^CD14^++^CD16^-^) was significantly higher than rPMLC (HLA-DR^+^CD14^++^CD16^+^) in BAL fluid (expressed as mean (SD) % of total PMLC in BAL fluid, n = 14; iPMLC 91.2 (3.9); rPMLC 8.8 (3.9); *P* < 0.0001, unpaired *t*-test) (Table 2).

**Table 2:** Cellular composition of cells present in BAL fluid

**Cell type**	**Proportion present in BAL fluid (mean (SD) % total cells)**
PMLC	17.8 (7.2)
Alveolar macrophages	26.4 (11.0)
Neutrophils	23.2 (11.0)
Lymphocytes	20.0 (10.5)

Data are expressed as mean (SD) % of total cells in BAL fluid (n = 14).

### Macrophage phenotype and proliferative capacity of PMLC subpopulations

Polychromatic flow cytometry was used to assess the phenotype and proliferative capacity of PMLC subpopulations in BAL fluid. In comparison with rPMLC, iPMLC had a significantly lower expression of the proliferation marker, Ki67, the transferrin receptor, CD71 and the macrophage mannose receptor, CD206. The scavenger receptor, CD163 and the mature macrophage marker, 25 F9, were expressed by a lower proportion of iPMLC compared to rPMLC, although this was not significant (Table 3).

**Table 3:** Cell surface antigen expression by PMLC subpopulations in BAL fluid following LPS inhalation

**ANTIGEN**	**EXPRESSED BY**	**rPMLC**	**iPMLC**
**Ki67**	Proliferating cells	19.0 [18.5 – 42.1]	2.1 [0.7 – 8.4]**
**CD71 (transferrin receptor)**	Mature macrophages	20.3 [15.7 – 31.7]	2.8 [1.5 – 3.6]***
**CD206 (mannose receptor)**	Alternatively activated macrophages	86.7 [72.8 – 97.9]	61.9 [40.6 – 73.9]*
**CD163**	Alternatively activated macrophages	92.2 [56.4 – 98.5]	69.6 [29.1 – 91.9]^NS^
**25 F9**	Mature macrophages	89.8 [73.1 – 97.0]	57.3 [14.4 – 97.5]^NS^

Data are expressed as median [interquartile range (IQR)] % of cells expressing each marker; n = 12; statistical analysis by Mann Whitney *U* test; ^NS^non-significant, *P < 0.05, **P < 0.005, ***P < 0.0005.

### Phagocytosis

**Figure 3:**
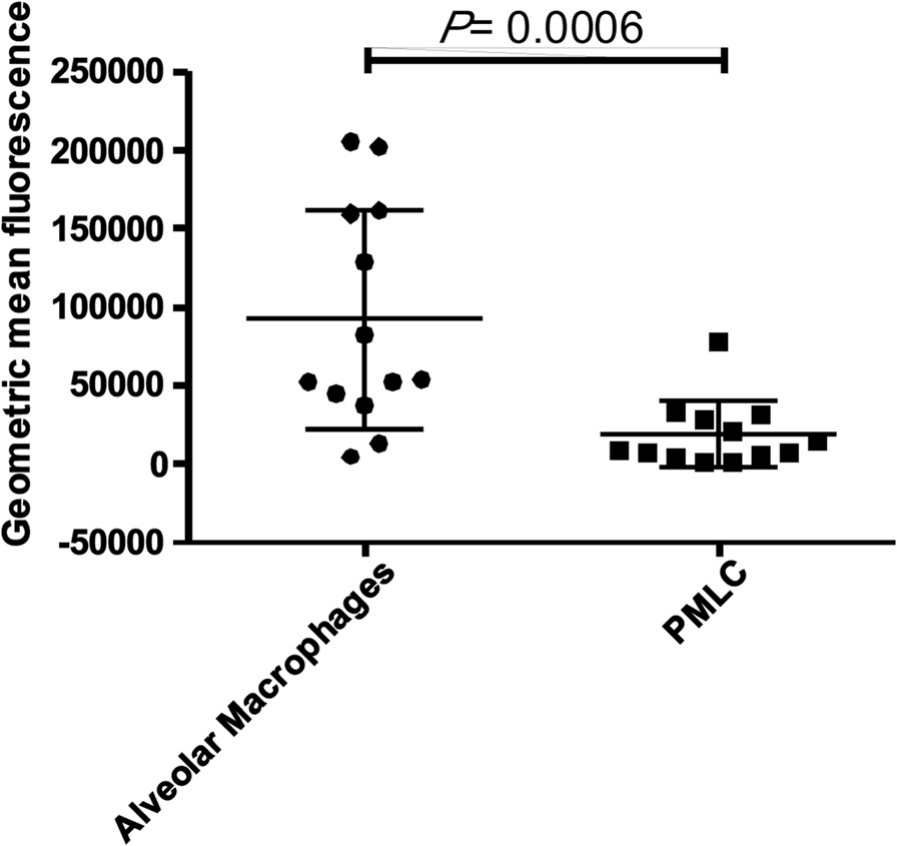
**Phagocytosis by cells in BAL fluid.** Alveolar macrophages displayed a significantly increased capacity for phagocytosis compared to PMLC (P =0.0006 by Mann Whitney *U* test (n = 13)).

Alveolar macrophages had a significantly increased ability to undergo phagocytosis compared to PMLC in BAL fluid (Figure 3).

### Cytokine production and response to dexamethasone by iPMLC and AM

**Figure 4:**
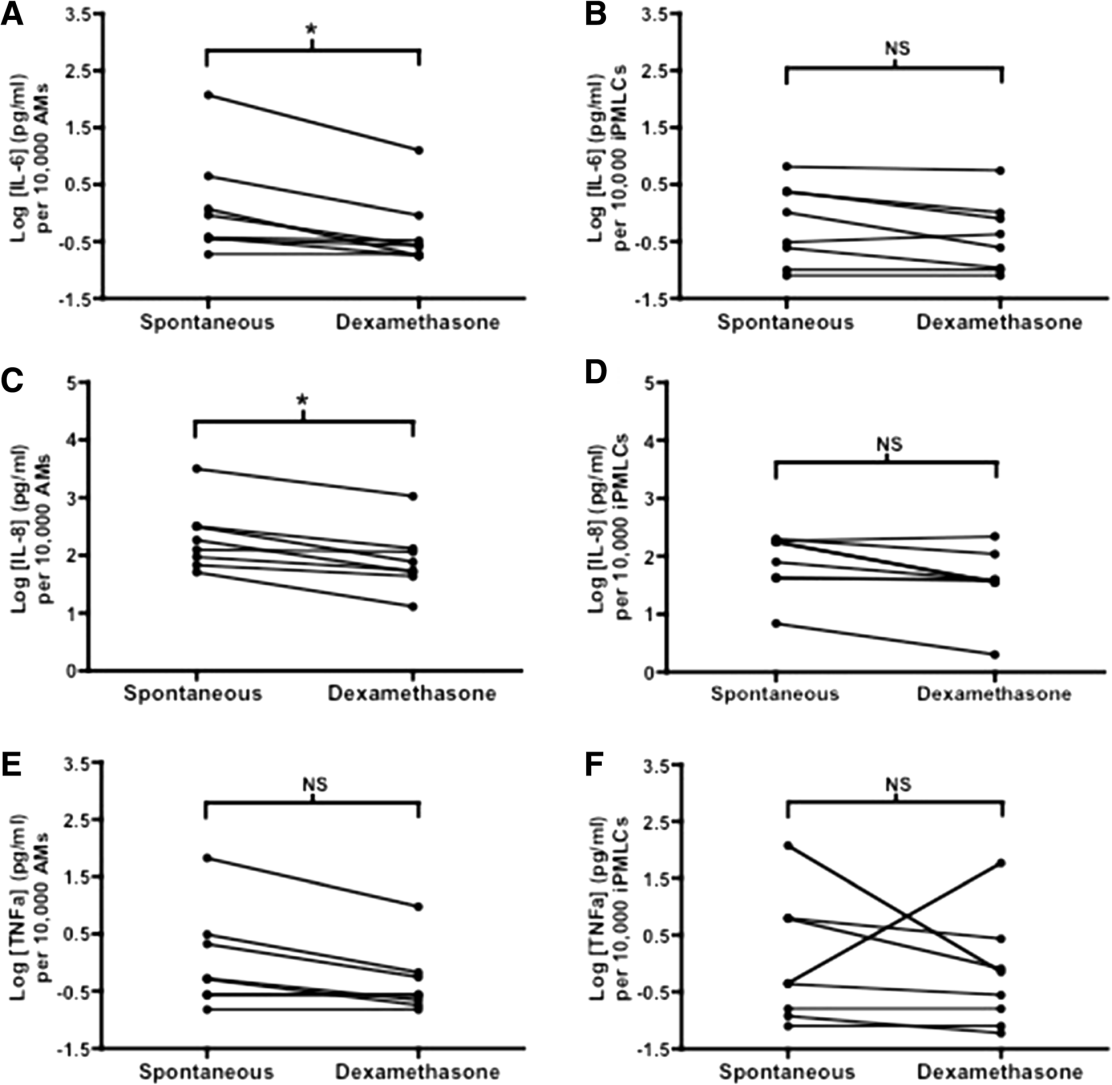
***In vitro *cytokine production by AM and iPMLC in response to treatment with dexamethasone.** Log concentrations of IL-6 **(Panels A-B)**, IL- 8 **(Panels C-D)** and TNFα **(Panels E-F)** in supernatants from flow-sorted, cultured AMs and iPMLCs. Data are shown paired (with/without incubation with dexamethasone) per 10,000 cells cultured; n = 8; statistical analysis was by Wilcoxon signed rank test; *P < 0.05.

iPMLC and AM were isolated from BAL fluid with high purity using FACS (Figure 2). Cells were grown in standard cell culture conditions with or without dexamethasone treatment (100 nM for 24 hours). There was no significant difference in spontaneous median interleukin (IL)-6, IL-8 or tumour necrosis factor-alpha (TNFα) production (pg cytokine per 10,000 cells) by iPMLC compared to AM, following isolation from BAL fluid and *in vitro* growth for 24 hours (n = 8, Figure 4). However a trend towards a difference in responsiveness to corticosteroid treatment was observed. Dexamethasone significantly suppressed IL-8 and IL-6 secretion by AM, with a trend towards suppression of TNFα secretion. In contrast, dexamethasone did not significantly suppress cytokine secretion by iPMLC after 24 hours of treatment (Figure 4).

## Discussion

Measures of LPS-induced inflammation were broadly consistent with our previous study, where subjects inhaled LPS but did not undergo active/sham leukapheresis [2], and therefore it is unlikely that cells present in BAL fluid of the subjects involved in this study were altered or activated, or that measures of inflammation were significantly changed by sham leukapheresis. Sham leukapheresis has been shown to have no effect on monocyte chemotaxis or migration [7].

Our original classification of inducible and resident PMLC subpopulations was based upon the variable expression of the monocyte markers CD14 and CD16, and the clear shift in the ratio of these cells in LPS-mediated experimental acute lung inflammation. We previously postulated that iPMLC may be sequestered from the circulation alongside neutrophils during self-limiting lung inflammation and, in contrast, that rPMLC were endogenous to the lung and contributed to macrophage homeostasis i.e. by undergoing proliferation and macrophage differentiation [2]. Our observation in the current study that rPMLC have a significantly increased expression of the mature macrophage markers CD206 (mannose receptor), CD71 (transferrin receptor) and 25 F9 (mature macrophage marker) and a significantly increased expression of the proliferation antigen Ki67, compared to iPMLC, is broadly supportive of our previous hypothesis, although this cannot be proven definitively, given the limitations and the nature of the current study. Conversely, iPMLC display a less mature, monocyte-like phenotype and have a significantly lower proliferative capacity compared to rPMLC. Therefore, it seems unlikely that the primary function of iPMLC is to contribute to regenerative demand and cellular turnover, and our data suggest that a pro-inflammatory function is more likely attributable to these cells, at least at the time point studied. This is supported by our cytokine data.

There are a number of limitations to this study that merit discussion. These principally stem from two problems. Firstly, flow sorting of cells from complex inflammatory populations in the human lung inevitably yields a relatively small and finite number of cells for study. Secondly, repeat bronchoscopy on the same day raises considerable ethical issues, and bronchoalveolar lavage itself leads to an inflammatory response in the lung after the procedure – these considerations inevitably meant that we could only study one time point in the process, and no inferences can be made regarding the time course and dynamics of any of the processes studied.

As such, due to the low numbers of rPMLC present in BAL fluid in this model it was not possible to isolate these cells by FACS in order to determine their basal cytokine production or response to steroid treatment, although this would be of interest for future studies. Due to the technical complexities associated with using FACS to isolate highly pure cell subpopulations from BAL fluid for *in vitro* growth and treatment with dexamethasone (i.e. samples of less than 95% purity were discarded), it was only possible to grow sorted cell populations from 8 of out 14 subjects. Notably, we were still able to demonstrate a significant response by AM to dexamethasone treatment, whereas iPMLC did not show any response to dexamethasone. The phagocytic capacity of individual PMLC subpopulations is of interest, although it was not possible to determine this due to limitations in the assay conditions. It is important to highlight that, despite significant differences in cell surface antigen expression by iPMLC and rPMLC, a degree of heterogeneity exists within both PMLC subpopulations, and it is plausible that functional crossover may occur. We have previously reported differential cell counts from cytocentrifuge preparations of BAL fluid from subjects who have inhaled LPS and then undergone active leukapheresis compared to those receiving sham leukapheresis [1], which was in accordance with the secondary endpoint of our RCT of pulmonary neutrophilia in BAL fluid. A discrepancy exists in the reported cellular composition of BAL fluid when comparing data from cytocentrifuge preparations compared to lineage analysis using polychromatic flow cytometry reported herein. However, we have shown that PMLC subpopulations are similar to lymphocytes and neutrophils in size, and it may be difficult to consistently distinguish PMLC from other lineages using cytocentrifuge preparations alone. Our data show that PMLC represent a significant proportion of the cells present in BAL fluid following LPS inhalation, similar in number to neutrophils at 9 hours. This observation was unexpected and implies that PMLC may play a significant role in the acute inflammatory response. It also implies that future studies of the cell content of BAL fluid should consider using flow cytometry to quantify the cellular composition of BAL fluid, taking into account PMLC subpopulations, according to the protocols described herein.

Experimental inhalation of LPS models some of the early features of acute lung injury (ALI) such as neutrophilia in peripheral blood and in the alveolar space, and protein leak across the alveolar-capillary membrane. The use of corticosteroids to treat ALI, and the optimal timing of administration, remains extremely controversial [8-11], and we felt it would be relevant to assess corticosteroid-responsiveness in distinct cell populations isolated from BAL fluid in volunteers who had inhaled LPS. iPMLC produced comparable concentrations of the inflammatory cytokines IL-6, IL-8 and TNFα compared to AMs, yet displayed a significantly reduced responsiveness to dexamethasone. Our data suggest that corticosteroids may potentially down-regulate secretion of inflammatory cytokines from AMs, but are unlikely to influence cytokine release from iPMLC. However, as discussed previously, the time point selected for study could have influenced these results. The BAL cells were collected at 9 hours after inhalation of LPS and the addition of dexamethasone was thus commenced at a relatively late stage; it remains possible that differences in dexamethasone-induced suppression of cytokine release observed between AM and iPMLC reflects a difference in the timing of peak cytokine release by both cell types. Corticosteroids inhibit apoptosis of neutrophils [12], the predominant alveolar cell in ALI, and this has been proposed as a potential reason for lack of therapeutic efficacy in this setting. In the current study we did not have sufficient cells to determine whether corticosteroids directly affect apoptosis of AMs and iPMLCs, and we recognise that any such effect could influence our findings.

In summary, our data show that after LPS challenge iPMLC constitute a significant proportion of the cellular component of BAL fluid. iPMLC have a low capacity for proliferation or phagocytosis and are capable of production of similar, albeit low, levels of pro-inflammatory cytokines compared to AM. Interestingly, unlike AM, iPMLC appear to be unaffected by corticosteroid treatment *in vitro*, at least in our experimental system. Further data are clearly required to understand the physiological or pathological relevance of these cells, and to determine whether their function and phenotype change as the inflammatory process proceeds. Nevertheless, we believe our findings represent a useful first step, suggesting the potential for iPMLC to play a role in the early regulation of inflammatory responses in the human lung.

## Abbreviations

ALI: Acute lung injury; AM: Alveolar macrophages; APC: Allophycocyanin; BAL: Bronchoalveolar lavage; BSA: Bovine serum albumin; FACS: Fluorescence-activated cell sorting; FEV1: Forced expiratory volume-1; FITC: Fluorescein isothiocyanate; FMO: Fluorescence minus one; IL: Interleukin; iPMLC: Inducible pulmonary monocyte like cells; IQR: Interquartile range; LPS: Lipopolysaccharide; PBS: Phosphate buffered saline; PE: Phycoerythrin; PMLC: Pulmonary monocyte like cells; RCT: Randomised controlled trial; rPMLC: Resident pulmonary monocyte like cells; SD: Standard deviation; TNFα: Tumour necrosis factor-alpha.

## Competing interest

The authors declare that they have no competing interest.

## Authors’ contributions

MB, LCB, ACM, JM, FR, SJ, AGR, KD, NH and AJS designed the study; MB, LCB, AJS coordinated the overall conduct of the study; MB, LCB, ACM, RD, FR, SJ acquired the data; MB, LCB, NA; AJS undertook data analysis and interpretation; MB, LCB, ACM, AJS drafted the manuscript; all authors approved the manuscript before submission.
